# Dynamic control of modeled tonic-clonic seizure states with closed-loop stimulation

**DOI:** 10.3389/fncir.2012.00126

**Published:** 2013-02-06

**Authors:** Bryce Beverlin II, Theoden I. Netoff

**Affiliations:** ^1^Department of Physics, University of MinnesotaMinneapolis, MN, USA; ^2^Department of Biomedical Engineering, University of MinnesotaMinneapolis, MN, USA

**Keywords:** seizure model, deep brain stimulation, tonic-clonic, synchrony

## Abstract

Seizure control using deep brain stimulation (DBS) provides an alternative therapy to patients with intractable and drug resistant epilepsy. This paper presents novel DBS stimulus protocols to disrupt seizures. Two protocols are presented: open-loop stimulation and a closed-loop feedback system utilizing measured firing rates to adjust stimulus frequency. Stimulation suppression is demonstrated in a computational model using 3000 excitatory Morris–Lecar (M–L) model neurons connected with depressing synapses. Cells are connected using second order network topology (SONET) to simulate network topologies measured in cortical networks. The network spontaneously switches from tonic to clonic as synaptic strengths and tonic input to the neurons decreases. To this model we add periodic stimulation pulses to simulate DBS. Periodic forcing can synchronize or desynchronize an oscillating population of neurons, depending on the stimulus frequency and amplitude. Therefore, it is possible to either extend or truncate the tonic or clonic phases of the seizure. Stimuli applied at the firing rate of the neuron generally synchronize the population while stimuli slightly slower than the firing rate prevent synchronization. We present an adaptive stimulation algorithm that measures the firing rate of a neuron and adjusts the stimulus to maintain a relative stimulus frequency to firing frequency and demonstrate it in a computational model of a tonic-clonic seizure. This adaptive algorithm can affect the duration of the tonic phase using much smaller stimulus amplitudes than the open-loop control.

## Introduction

Approximately one third of patients with epilepsy do not have sufficient control of their seizures even with the use of antiepileptic drugs. The use of deep brain stimulation (DBS) to suppress or truncate seizures is an alternative approach for controlling seizures in drug refractory patients. However, DBS for seizure suppression has had mixed clinical success (Loddenkemper et al., 2001). The SANTE trial, a multi-center clinical trial, used open-loop DBS, and demonstrated a 35% reduction in seizures (significantly more than in the control group), but with very few seizure free patients (Fisher et al., 2010). Neuropace has developed a closed-loop stimulator that has been tested in multi-center clinical trials, resulting in a 37.9% decrease in seizures, which is also significant compared to a control group. Although some patients are reluctant to have a device implanted in their brain (Arthurs et al., 2010), there exists a population of patients who have exhausted other medical options and are willing to take surgical risks for any reduction in seizures. There is therefore a need to improve the efficacy of DBS.

We presume that DBS may be more effective if the stimulation parameters could be optimally tuned for each patient. However, improving the efficacy of DBS by tuning the stimulus parameters is difficult, particularly as the mechanism by which DBS suppresses seizures is poorly understood (Vonck et al., 2003). With a better understanding of the mechanisms by which DBS functions, we may be able to design and optimize stimulus parameters and develop a closed-loop stimulator that tunes the stimulus parameters. This paper illustrates how DBS stimulus parameters can be selected based on the dynamics of neurons within the targeted brain area in order to affect synchrony in different stages of a seizure.

There are several different working hypotheses about the underlying mechanism by which DBS is able to suppress seizures. In animal models indirect evidence suggests that stimulation in the anterior thalamic nuclear complex can induce a release of the inhibitory neurotransmitter GABA, which presumably depresses the activity of neurons and results in the observed increase of seizure threshold (Mirski et al., 1997). In brain slice experiments it is possible to directly measure the effect of DBS stimuli in neurons. It has been shown that high frequency stimulation can cause neurons to go into a depolarization blockade, where cells are unable to fire, that will truncate the seizure (Bikson et al., 2001). DC electric fields can be used to hyperpolarize neurons, in order to change the neuron's excitability and suppress seizures (Gluckman et al., 1996). It has also been suggested that the stimulation may prevent neuronal synchronization; under this hypothesis DBS stimuli with a Poisson train of pulses at the same frequency as the high frequency stimulation has been shown to suppress seizures (Wyckhuys et al., 2010).

### Tonic-clonic seizures

Grand-mal epileptic seizures consist of two major stages: the tonic and clonic phases. In the tonic phase patients lose consciousness and their muscles tense up, while in the clonic phase the patients begin to jerk (Fisch and Olejniczak, 2006; Bragin et al., 2010). High frequency oscillations (HFOs) (oscillations above 150 Hz) in the intracranial electroencephalogram (EEG) recordings are observed during these seizures (Schindler et al., 2007b) as well as using magnetoencephalogram (MEG) (Garcia Dominguez et al., 2005; Perez Velazquez et al., 2007). In human studies, it has been shown that firing rates at the onset of the seizure are very high and decrease over the course of the tonic-clonic seizure (Ward, 1961). EEG measurements suggest that population amplitude and coherence is greater in the clonic phase than the tonic phase (Quian Quiroga et al., 1997). Frequency sweeps observed in EEG during seizures are a biomarker that can be used to detect seizures (Schiff et al., 2000).

We have recently proposed a model explaining (1) a mechanism for the transition from tonic to clonic phases by slowing of firing of neurons over the seizure and (2) the differing ability of neurons to synchronize at high firing rates and low firing rates (Beverlin et al., 2011). The change in the firing rate in the model is due to due to synaptic depression of the neurons and spike rate adaptation of the neurons, both of which occur at high firing rates (Abbott et al., 1997; Manor and Nadim, 2001). When analyzing EEG, determining the transition between tonic to clonic is somewhat subjective to the epileptologist. In this paper we will simply define the tonic phase of the seizure as network activity with high firing rate and low synchrony, while the clonic phase is high firing rate with high synchrony.

### Firing rate and network synchrony

In previous brain slice experiments, it was found that while the firing rates of neurons were high during the tonic phase of the seizure, neurons exhibited a low degree of correlation. During the clonic phase the firing rate decreased and the population became highly synchronous (Netoff and Schiff, 2002). This transition from the tonic to clonic phases may be integral to the evolution of the seizure and its eventual termination. Based on this hypothesis, it has been shown that synchronizing populations with DBS pulses may promote seizure termination and truncate the seizure (Schindler et al., 2007a). We have recently developed a computational model that illustrates a mechanism by which synchrony changes during a seizure (Beverlin et al., 2011).

In our model, seizures start by the failure of inhibition. Without inhibition, the excitatory neurons increase their firing rate and excitatory drive within the network increases in a positive feedback loop resulting in very high firing rates. Over time, the firing rate slows down, and the network transitions to a synchronous high amplitude clonic phase of seizure. In the model the transition from tonic to clonic phases is caused by a change in the sensitivity of neurons to synaptic inputs as their firing rate slows; this leads to a shift in synchrony. We demonstrate how the transition occurs in a network of model neurons and explain the mechanisms using pulse-coupled oscillator theory. There are several ways in which network synchrony may change *in vivo*, including the reintroduction of activity from the inhibitory population (provided they have entered depolarization block at the seizure onset) (Ziburkus et al., 2006), synaptic depression, and vesicle depletion. In our model, the change in firing rate is produced by including synaptic depression and a gradually decreasing input current to the model neurons, to simulate spike rate adaptation of the neurons.

### Periodic stimulation in epilepsy

DBS has been tested in models of epilepsy in order to disrupt seizures (Good et al., 2009; Fisher et al., 2010; Nelson et al., 2011; Rajdev et al., 2011). Stimuli designed to increase synchrony has been shown to effectively truncate seizures (Schindler et al., 2007a) and DBS has been employed in clinical trials with reasonable success (Morrell, 2006; Fisher et al., 2010; Morrell and On behalf of the RNS System in Epilepsy Study Group, 2011).

During a seizure the firing rate of neurons changes as the phases of the seizure progress. Therefore, we hypothesize that the influence of DBS on population synchrony will change if the stimulus does not adapt to the firing rate of the neuron. In this paper we first estimate the effects of periodic DBS on neuronal populations that are firing at high rates during the tonic phase, and then on the low firing rates during the clonic phase. To study the effects at each phase of the seizure, we fix the synaptic strengths and apply constant current and vary the DBS frequency and amplitude measuring the resulting increase or decrease in synchrony. We find that independent of firing rate, there are ratios of stimulus frequency to neuronal frequency that can either synchronize or desynchronize. Then, in the full model with changing firing rates, we demonstrate an adaptive algorithm that measures the firing rate of a neuron to adjust the stimulus frequency to maintain stimulation in the regime that promotes or decreases synchrony over the entire duration of the seizure. This exemplifies how an adaptive stimulus algorithm may be designed to disrupt synchrony in a population where the population oscillation is changing.

## Methods

We investigate the effectiveness of DBS within an epileptic model using computational simulations of excitatory neuronal networks. The neuron model captures the dynamics of a real neuron's sensitivity to synaptic inputs, current inputs, and periodic forcing from applied stimuli. Synaptic depression variables change the recurrent excitatory drive amongst the population, which changes the firing rates of the neurons. As the neuron's firing rate changes, the sensitivity to synaptic inputs also changes, allowing them to synchronize at slow firing rates, but not at high firing rates. Networks of neurons are connected using a second order network (SONET) that keeps the neurons at the edge of synchrony at the high firing rate.

### Morris–lecar model neuron

We use a modified version of the Morris–Lecar (M–L) model neuron (Morris and Lecar, 1981; Izhikevich, 2007), a 2-D reduction of the Hodgkin–Huxley model (Rinzel, 1985). DBS stimulation is simulated by applying periodic pulses of current input of varying strength and frequency, depending on the stimulation protocol. The conductance based M–L model calculates the change in voltage as a function of the membrane's ionic currents as described by the following equations:

$$\begin{array}{l} \;C\dot{V}=I_{input}+I_{DBS}+I_{noise}-g_{L}(V-E_{L})\\ \;-\;g_{Na}m^{\infty }(V)(V-E_{Na})-\;g_{K}n(V-E_{K})\\ \;-D(S-F)(V-E_{syn}),\\ \;\dot{n}=\frac{n_{\infty }(V)-\;n}{\tau (V)}\\ m_{\infty }(V)=\;\frac{1}{1+\;e^{\frac{V_{1/2}-\;V}{k}}}\;\\ \;\tau (V)=\;C\;e^{\frac{-{\left(V_{max}-V\right)}^{2}}{\sigma^{2}}}\\ \;\dot{S}=-\frac{S}{\tau_{s}}\overset{j\;=\;1}{\underset{M}{\sum }}\delta \text{​}(t-t_{syn}^{j})\\ \;\dot{F}=-\frac{F}{\tau_{f}}\overset{j\;=\;1}{\underset{M}{\sum }}\delta \text{​}(t-t_{syn}^{j}) \end{array}$$

where *C* is the membrane capacitance, *V* is the membrane voltage, *I*_input_ is an input current common to all neurons, *I*_noise_ is a white noise input proportional to the square root of the time step independent to each neuron, *g* are the maximal conductances of each current source, *E* are the reversal potentials for each ion, *m* and *n* are the ionic gating variables, where *m*_∞_ and *n*_∞_ are the steady-state activation for a given voltage, *V*_1/2_m satisfies *m*_∞_ (*V*_1/2_) = 0.5, *V*_max_ is the value of *V* at the maximum value of *m, k* is the degree of slope at *V*_1/2_, τ is the voltage dependent time constant of the inactivation variable, σ determines the sensitivity of the time constant of *V, S* represents the slow variable of the synaptic input shape, with a time constant τ_s_ and *F* is the fast synaptic time constant. At times of synaptic input, 1 is added to both the *S* and *F* state variables for each presynaptic event at time *t*_syn_ for all *M* events. Synaptic depression, *D*, is defined as *D*_*i* + 1,*j*_ = *D*_*i,j*_*d*, updated for cell *j* after a synaptic input *i* as described in Varela et al. (1997) where the strength of depression is controlled by *d*.

The model is explained in more detail in our recent seizure model paper (Beverlin et al., 2011). The parameters of the M–L model were chosen so that the phase response curve (PRC) is similar to PRCs we have measured in hippocampal pyramidal neurons (Netoff et al., 2005); they are as follows: *C* = 1.0 μF, *g*_L_ = 8 nS, *E*_L_ = −53.24 mV, *g*_Na_ = 18.22 nS, *E*_Na_ = 60 mV, *g*_K_ = 4 nS, *E*_K_ = −95.52 mV, *V*_1/2 m_ = −7.37 mV, *k*_m_ = 11.97 mV, *V*_1/2n_ = −16.35 mV, *k*_n_ = 4.21 mV, τ = 1 ms, spikeWidth = 0.03, *E*_syn_ = 0, τ_f_ = 0.25 ms, τ_s_ =0.5 ms. Matlab code for this model is available at http://neuralnetoff.umn.edu/public/TonicClonicControl and from Model DB website (http://senselab.med.yale.edu/modeldb).

### Network structure

**Figure 1:**
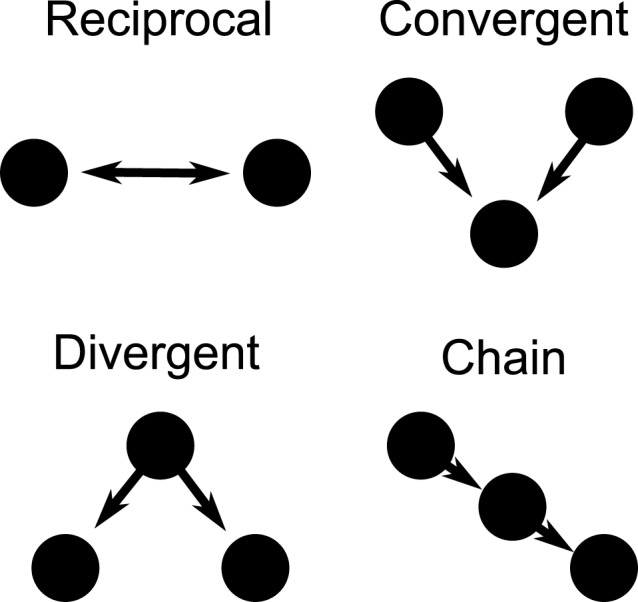
**Second order motifs.** Connection motifs of two and three-cell combinations with two directional connections. The motifs are reciprocal, convergent, divergent, and chain motifs. The prevalence of these motifs within a larger network can be specified when generating the network.

Directional networks of 3000 cells were generated with an average of 30 out-going excitatory synaptic connections using a second order network topology (SONET), which places additional correlated structure to random networks (Zhao et al., 2011). The specific network structure is determined by specifying the average connectivity (first order statistic) as well as the additional prevalence of two edge motifs, thus referred to as second order motifs. These second order structures are reciprocal, convergent, divergent, and chain connections, as illustrated in Figure 1. We generate large networks by specifying the first and second order statistics. It has been found that the prevalence of chains and convergent connections have a strong effect on the synchronizability of the network. Here we choose the network statistics which allow a network to both synchronize and desynchronize, depending on input current and firing rate. The network we use has statistics similar to that measured in rat visual cortex (Song et al., 2005). The specific SONET was chosen out of 186 candidates discussed in recently published results (Zhao et al., 2011). This network, which had 4 times the prevalence of reciprocal connections, 1.4 times the convergent connections, 1.3 times the divergent connections, and 1.2 times the chain connections compared to a random graph, was the closest to measured cortical networks in a rat model.

### Network synchrony measure

Network synchrony is quantified using the Kuramoto order parameter (*r*) which ranges from 0 (neurons evenly distributed in phase) to 1 (neurons in coherent phase) and calculated as follows:

$$re^{i\phi }=\frac{1}{N}\sum_{j=1}^{N}e^{i\theta_{j}}$$

where the phases of neurons (θ_*j*_) are summed to create a population vector with magnitude (*r*) (Kuramoto, 1984; Strogatz, 2000).

## Results

**Figure 2:**
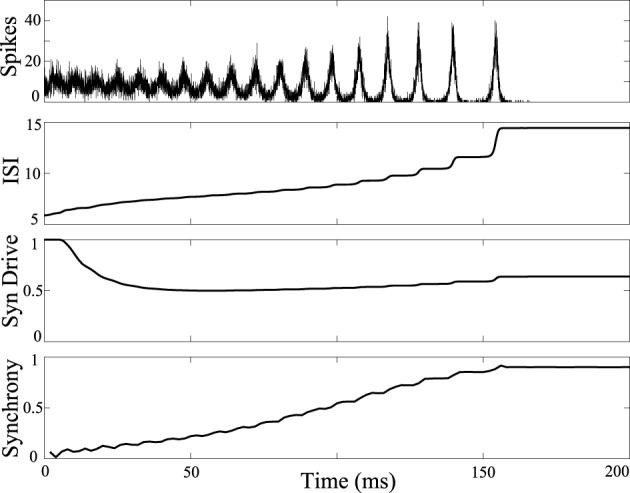
**Tonic-clonic transition in model network.** Synchrony emerges in large scale networks with synaptic depression and ramped current from *I* = 0nA to *I* = −5nA and inclusion of synaptic depression. Average synaptic strength across the population is plotted against time in the 3rd panel labeled as “Syn Drive.” Synchrony as a function of time is plotted at the bottom, measured using the Kuramoto order parameter. Synchrony emerges as interspike intervals of the neurons exceed about 7 ms. Synchrony will not occur if tonic current is held at 5 nA keeping interspike intervals shorter than about 7 ms.

The influence of DBS was tested in a network model that reproduces a tonic to clonic shift in network synchrony as a function of the firing rate of the neurons (Beverlin et al., 2011). In the simulations, at the seizure onset the firing rate of the neurons are very high, as might be expected with runaway excitation, and then over the duration of the seizure, the firing rate of the neurons slowly decreases, eventually bringing about a transition to the clonic phase of the seizure, seen in Figure 2. The tonic-clonic transition model reproduces the shift in synchrony observed in EEG. In this model, the firing rate was modulated by a combination of changes in tonic drive to all the neurons, representing drive of exogenous sources, and synaptic depression from neurons within the network. Simulations included 3000 M–L neurons connected using a second order network designed to be at the edge of synchrony when neurons were in the tonic phase of the seizure. Over the duration of the seizure we decreased the tonic drive to represent depression from the exogenous inputs, and the synapses within the network depress during the seizure due to the modeled synaptic depression. Decreased input from both the exogenous and endogenous sources results in a decrease in firing rate over the duration of the seizure.

In this paper we apply periodic stimulation to the seizure model. All cells receive the same stimulus input for a given set of stimulus parameters, assuming that the population is uniformly distributed from the electrode. To analyze the effects of the stimulation in each phase, we hold the applied current in the neurons constant and freeze the synaptic plasticity to study the effects of stimulation at each phase of the seizure separately. We analyze and model the effects at a high firing rate during the tonic phase and then again at a low firing rate during the clonic phase. Then, we restore the changing exogenous current and plasticity back into the model to measure the effects of periodic stimulation to the duration of the tonic and clonic phases.

### Open-loop periodic stimulation with fixed drive to neurons

**Figure 3:**
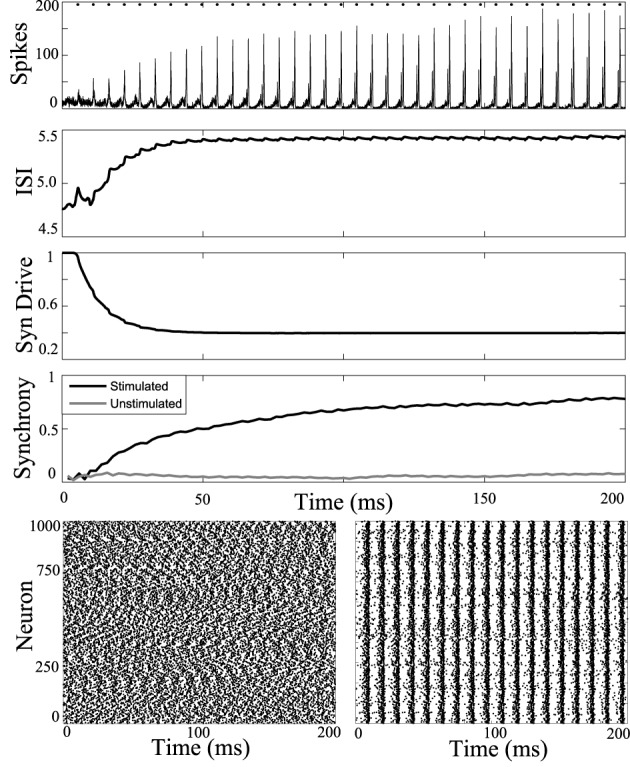
**Synchronizing a tonic phase model using periodic drive.** Computational simulation of network activity with current set to 6 nA to simulate tonic phase of seizure. **Top**, population is entrained to periodic stimuli (points of stimulation as dots along top axis). ISI increases from 4.8 to 5.5 ms. Synaptic drive decreases from depression due to strong input from DBS. Synchrony of unstimulated network is low (gray) and stimulating with 5.5 ms pulses increases synchrony. **Below**, rasterplots of neuronal network spike times during unstimulated tonic activity and with network stimulation to synchronize. Spike times of 1000 model neurons from the 3000 cell network simulation. **Left**, unstimulated cells have low synchrony. **Right**, network stimulated with 5.5 ms pulses becomes coherent.

First, periodic stimulation was applied to a network simulation driven with high current input (6 nA), to model the tonic phase of the seizure. At this high firing rate the unstimulated network does not synchronize. Results of stimulation applied to all cells of the network at 5.5 ms intervals are shown in Figure 3. Stimulus at this interval during the tonic phase increases synchrony in the tonic phase.

**Figure 4:**
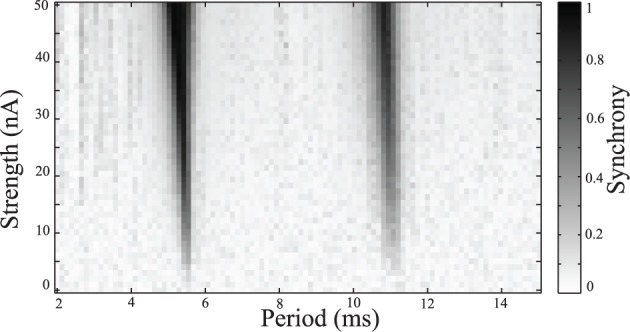
**Synchrony map of stimulated tonic networks.** Current input of 6 nA applied to all cells. Grayscale indicates calculated synchrony as the Kuramoto order parameter averaged over the last 200 ms of individual simulation for a range of stimulus amplitudes and periods.

Simulations were repeated while varying the stimulation frequency and amplitude. Synchrony was measured using the Kuramoto order parameter, averaged over the last one quarter of the simulation to estimate the steady-state synchrony in the network. These simulations were repeated over a range of stimulus amplitudes and frequencies, results are shown in Figure 4. Darker areas indicate stimulus parameters that entrain the neurons, resulting in a synchronized population. These entrained regions are known as *Arnold Tongues* (Milton and Jung, 2003). These “tongues” of entrainment occur at integer ratios of stimulus period to the natural period of oscillation. The points on the map that are lightly shaded indicate those parameters where the network remains desynchronized.

**Figure 5:**
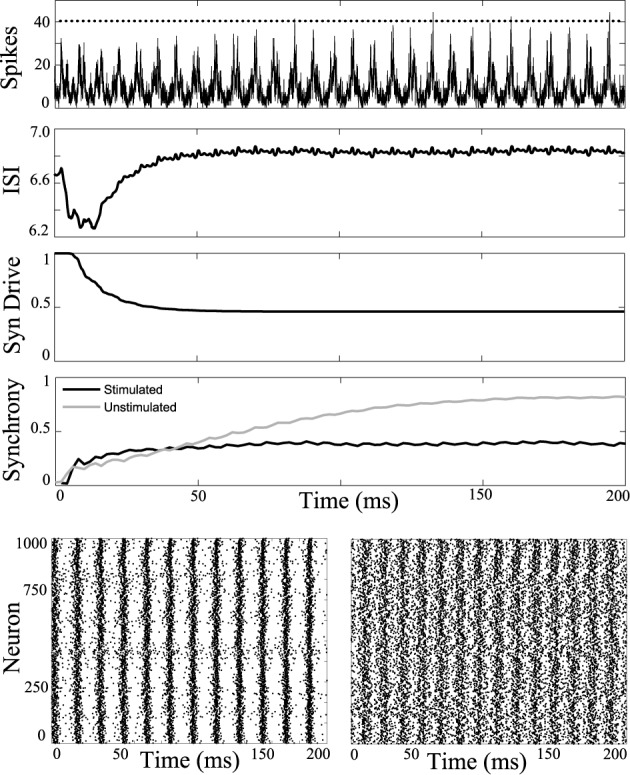
**Desynchronizing the clonic phase.** Computational simulation of network activity with current set to −2 nA to simulate clonic phase of seizure. See Figure 3 for general figure description. Synchrony of the unstimulated clonic network increases to a strong value near 0.8 (gray). When applying the 2 ms periodic DBS pulse, the network activity is desynchronized. Bottom Left, unstimulated cells have high synchrony. Bottom Right, network stimulated with 2 ms pulses. Less synchronous activity is observed in the stimulated network.

The simulations were then repeated while applying a −2 nA current, in order to simulate a network during the clonic seizure phase, shown in Figure 5, where the unstimulated network would spontaneously synchronize. The network is then periodically stimulated with a 2 ms period, shown as dots along the top curve in Figure 5. This stimulation reduces the synchrony compared to the unstimulated simulation.

**Figure 6:**
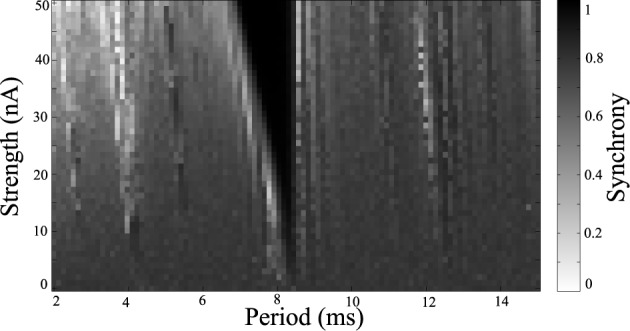
**Synchrony map of stimulated clonic networks.** Current input of −2 nA applied to all cells. Grayscale indicates calculated synchrony as Kuramoto order parameter averaged over the last 200 ms of each simulation for a range of stimulus amplitudes and periods.

Simulations were run for a range of stimulus amplitudes and frequencies, while driving the network at −2 nA. A synchrony map for these results is shown in Figure 6. One notable difference is that the region of entrainment has shifted from 5.5 ms around the natural period when the system is driven with 6 nA, to a region of entrainment of 8.5 ms around the natural period when driven at −2 nA. Because the low current network synchronizes spontaneously, a wider range of stimulus parameters synchronize the network. There are several windows which desynchronize the population. In the example shown in Figure 5, we use 2 ms period for stimulation, but 4 ms or about 12.5 ms for example could be used as indicated by light bands in Figure 6.

### Continuous control of seizures with variable stimulus frequency

Ultimately, control of seizure states may be most effectively achieved by implementing a closed-loop feedback system, in order to select the stimulus frequency from the measured neuronal frequency (Nelson et al., 2011). We have noticed that the stimulus frequency that entrains the neurons occurs at frequencies just slightly faster than the firing rate of the neurons. Stimulus regimes that desynchronize the population are found to be slightly slower than the firing rate of the neurons. Based on this observation, we developed a simple feedback system that modulates the stimulus period depending on the firing rate of one cell within the network. All other parameters are the same as previously used in the unstimulated tonic-clonic model, including the current ramp and network topology. Here, the feedback system selects the stimulus frequency based on a user chosen ratio of stimulus to measured frequencies. This ratio can be selected from the entrainment maps. A choice of 1:1, for example, would entrain a population, while a choice of 1:1.14 (stimulated to measured frequency ratio) may desynchronize a population.

**Figure 7:**
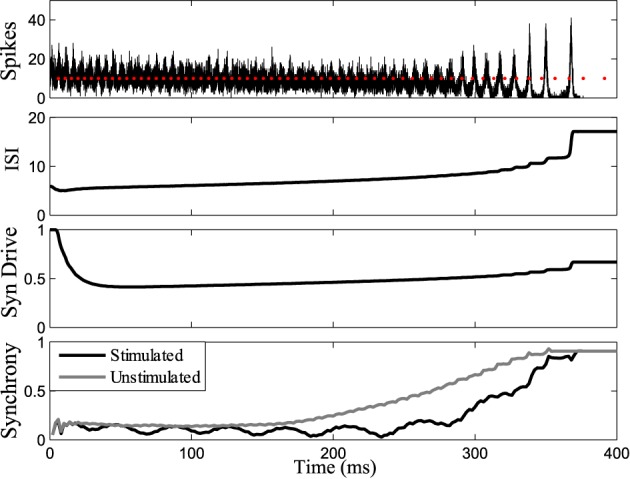
**Tonic phase prolonged.** Stimulating at a desynchronizing frequency ratio of 1:1.14 where the stimulus is firing slightly slower than the measured frequency of one chosen cell. The tonic phase of low synchrony is prolonged when stimulating compared to the unstimulated model. Graphs labeled the same as Figure 3.

**Figure 8:**
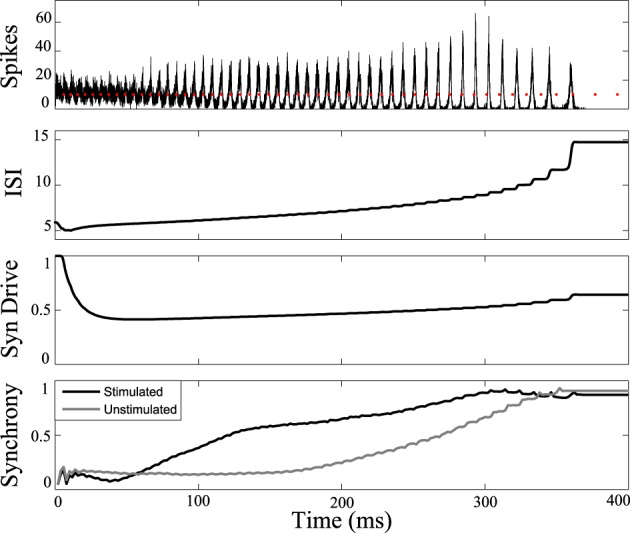
**Tonic phase truncation.** Network of cells stimulated at 1:1 frequency ratio compared to one measured cell in the network. Bottom: Transition to synchronous clonic phase is earlier (black line) than the unstimulated model (gray line). Here the clonic phase is extended. Graphs labeled the same as Figure 3.

Figure 7 shows the response of the network while stimulating at intervals 1.14 times the interspike intervals of neurons in the population. Synchrony emerges later in this stimulated case than the unstimulated network, prolonging the tonic phase. Eventually, the network slows sufficiently such that synchrony takes over, despite the dispersive effects of the stimulus. Conversely, by applying the stimulus at the same frequency as the firing rate of the neurons (1:1 ratio) we were able to bring about synchrony in much less time than in the unstimulated case, truncating the tonic phase, as shown in Figure 8. In both the synchronizing and desynchronizing closed-loop feedback experiments we used stimulus amplitude of 10 nA, one quarter the amplitude used in the open-loop conditions to achieve a similar effect.

## Discussion

Tonic-clonic seizures can be devastating to a patient with epilepsy. While there is evidence that DBS can reduce seizures, no clinical application has been found to be fully effective in truncating seizures. It is well known in oscillatory models that periodic forcing of a network of oscillators can synchronize or phase disperse the oscillators (Glass and Mackey, 1988; Elbert et al., 1994; Kaplan et al., 1996). It has previously been proposed that this may be used to control seizures (Milton and Jung, 2003). In a recent paper, we proposed that this may be involved in treating Parkinsonian symptoms (Wilson et al., 2011). In this paper we use similar periodic stimulation theory to affect the tonic and clonic phases of a seizure in a computational model we have recently developed (Beverlin et al., 2011). Recently we proposed that the shift from the desynchronized tonic phase to the synchronous clonic phase occurs as the neuronal firing rate adapts over the duration of the seizure. At the high firing rates, the model neurons do not synchronize, but as the firing rates slow down, the cells become more sensitive to synaptic inputs and the network synchronizes. The change in spike rate is modeled by gradually decreasing the current drive to the neurons along with depressing synapses.

In this paper, we have added periodic stimulation to the tonic-clonic model to determine if periodic stimulation could be used to affect the duration of the seizure phases. We analyzed the effects of stimulus frequency and amplitude on the population synchrony at the tonic phase and again at the clonic phase. Depending on the stimulus frequency we were able to synchronize neurons during the asynchronous tonic phase, or desynchronize neurons in the synchronous clonic phase. Periodic stimulation at integer ratios of the stimulus frequency to the natural frequency was found to entrain and thereby synchronize the population. Conversely, periodic stimulation just slightly slower than the firing rates (and at some frequencies, faster than the firing rates of the neurons) could desynchronize the population. Our findings can be explained with PRC theory, which we previously used to explain the effects of the stimulus at different frequency amplitudes and its effect on population synchrony (Beverlin et al., 2011). The effect of firing rate shifting the peak of the PRC to the left in response to excitatory inputs is generally true and should therefore not be heavily model dependent (Gutkin et al., 2005; Fink et al., 2011). We chose the M–L model because it is one of the simplest conductances based neuronal models that can demonstrate this effect.

Periodic stimulation of a network during a seizure with a fixed period would have a mixed effect; synchronizing at some phases of the seizure and desynchronizing at others, as the neurons are constantly changing their firing rate. However, the ratio of stimulus frequency to neuronal firing rate that entrains or desynchronizes the population is relatively consistent. Therefore, we created a closed-loop control system that adjusts the stimulus frequency to desynchronize or synchronize the population, holding the stimulus at a fixed rate relative to the neuronal firing rate. In this case the tonic phase of the seizure could effectively be shortened by applying a stimulus at the same frequency of the neurons, while the tonic phase could be prolonged by applying a stimulus frequency that is slightly slower than the firing rate of the neurons, effectively preventing the synchronization of the population.

This model illustrates the principle that periodic stimulation at certain ratios to the measured firing rate of neurons can be used to promote or decrease synchrony and this principle may be used in a closed-loop feedback system for seizure suppression. We are not suggesting that this model is an accurate model of the actual physiology in the brain. Instead, if PRCs can be measured during seizures, our theory may be tested experimentally. We plan to test these hypotheses in brain slice experiments in the near future.

In addition, the complicated structure and function of real neurons in real tissue are beyond the scope of this paper. Here we have investigated the applicability of DBS in a model network; naturally, there may be real world complications when implementing these protocols depending on the location of the electrode(s) and stimulus parameters. In addition, clinical applications of DBS thus far are typically less than 200 Hz. For example the SANTE trials studying the treatment of refractory epilepsy used a stimulation of 145 Hz (Fisher et al., 2010). Some of the frequencies in our model presented here exceed these typical frequencies, but the relative frequency between the stimulus and the neuronal firing rate is what we consider important. Our model is not designed to produce realistic firing rates, so we do not suggest based on this model that these are realistic stimulation frequencies for all brain regions that should be used clinically.

There are many aspects of this simulation which are not physiologically realistic which could be improved in future studies. First, the neurons are modeled as oscillators. Generally, neurons do not fire periodically. However, at the onset of a seizure with high rate of synaptic asynchronous synaptic inputs, neurons may fire close to periodically. All the neurons are also modeled as oscillators with the same parameters and the same firing rate, while it would be more realistic to model the neurons with a distribution of parameters and firing rates. Furthermore, in this model the stimulus was applied uniformly to all the neurons. In a real neuronal network there is geometry to the position of the neurons and a stimulus electrode will not uniformly stimulate all the neurons. All of these aspects of the model could be improved to make it more realistic, and will be the focus of further investigation, but we do not feel will change the fundamental approach we present here to desynchronizing populations.

How might this algorithm be implemented in practice, such as in a brain slice model of seizures and eventually in humans? First, a stimulation electrode and a recording electrode are needed. Then, it is necessary to determine the optimal stimulus frequency ratio with respect to the neuronal frequency. This can be determined from the neuron's PRC to the stimulus. The PRC is measured by open-loop stimulation at random intervals that are on average much longer than the period of the neuron on average. The phase of the oscillation is measured before and after the stimulation to estimate the phase advance of each stimulus. Generally, some model representing the phase advance as a function of the stimulus phase is fit to the resulting data. PRCs would need to be measured at different firing rates or phases of the seizure. From the measured PRCs the Lyapunov Exponents (LEs) of the population response at is estimated different stimulus amplitudes and frequencies (Wilson et al., 2011). Stimulus parameters are selected that maximize the LE to desynchronize the population, or minimize the LE to synchronize. To implement the algorithm, the recording electrode would be used to measure the firing rate of neurons in the population; the measured firing rate would then be used to modulate the frequency of the stimulating electrode.

An interesting finding is that the closed-loop controller could affect the duration of the tonic phase with equal efficacy at one quarter the stimulus amplitude than the open-loop control. This indicates that a simple measure of the neuronal firing rate may significantly improve the efficacy of DBS.

It is important to note that we do not propose that it is best to synchronize and shorten the duration of the tonic phase of the seizure, or to prolong it. We consider that the restructuring of the neuronal network by induction of synaptic plasticity by high firing rates of neurons during seizures may ultimately be the long term deleterious effect if seizures. The goal of the therapy may be to minimize the plasticity changes during a seizure. If neurons fire synchronously, plasticity may be greater than when neurons fire asynchronously. In this case, maximizing the tonic phase of the seizure and minimizing the clonic phase may result in less plasticity changes. However, if the synchronization of the population is integral to the termination of the seizure promoting synchrony may terminate the seizure earlier (Schindler et al., 2007a). For example, if seizures are sustained by recurrent excitation, increasing synchrony may decrease the excitable pool of neurons, thereby decreasing the likelihood of re-entry and terminating seizures earlier. Using a stimulus that can modulate the duration of the tonic phase may help us determine whether synchrony is just a network behavior that occurs at the termination of the seizure or whether it is integral to the termination.

HFOs are population oscillations that are seen between seizures. Suppressing these oscillations may be considered a target for DBS stimulation. The hope would be that disrupting these pathological oscillations may suppress epileptogenesis. The same approach used in this paper might be used to design a stimulus to suppress HFOs. HFOs might be a good target because they are observed to increase prior to a seizure in human and animal models (Worrell et al., 2004), and are thought to arise from synchronous bursts of neurons that occur in an epileptic focus (Bragin et al., 1999, 2010; Ibarz et al., 2010). There is also strong experimental evidence that synchrony amongst cortical regions is increased in epileptic patients (Bullock et al., 1995; Towle et al., 1999; Ben-Jacob et al., 2007; Schevon et al., 2007; Prusseit and Lehnertz, 2008; Zaveri et al., 2009) and that this synchrony changes in the lead up to a seizure (Lehnertz and Elger, 1995; Chavez et al., 2003; Le Van Quyen et al., 2005). In contrast, other evidence suggests that synchrony may decrease prior to a seizure (Mormann et al., 2003). We hypothesize that tuning DBS stimulators to desynchronize prominent pathological oscillations relevant to the generation of seizures interictally suppress seizures. However, we are not aware of any direct evidence that DBS affects these oscillations.

## Conclusion

This work proposes a novel method to alter seizures using DBS. In a computational model we have demonstrated that the duration of the tonic-phase of a seizure may be extended or shortened by promoting synchrony using periodic stimulation. Promoting or decreasing synchrony depends on the relative frequency of the stimulation to the firing rate of the neurons. By using a closed-loop feedback to adjust the stimulation frequency dependent on the firing rate of the neurons, we are able to extend or decrease the duration of the tonic phase with much weaker stimulus pulses than was necessary in open-loop stimulation.

### Conflict of interest statement

The authors declare that the research was conducted in the absence of any commercial or financial relationships that could be construed as a potential conflict of interest.
